# High-Temperature Spin Crossover in Iron(II) Complexes with 2,6-Bis(1*H*-imidazol-2-yl)pyridine

**DOI:** 10.3390/molecules27165093

**Published:** 2022-08-10

**Authors:** Ludmila G. Lavrenova, Olga G. Shakirova, Evgeniy V. Korotaev, Svetlana V. Trubina, Alexsei Ya. Tikhonov, Irina A. Os’kina, Sergey A. Petrov, Konstantin Yu. Zhizhin, Nikolay T. Kuznetsov

**Affiliations:** 1 Nikolaev Institute of Inorganic Chemistry, Siberian Branch, Russian Academy of Sciences, Academic Lavrentyev Avenue 3, 630090 Novosibirsk, Russia; 2 Department of Chemistry and Chemical Technologies, Faculty of Machinery and Chemical Technologies, Federal State Budget Institution of Higher Education, Komsomolsk-na-Amure State University, Lenin Avenue 27, 681013 Komsomolsk-on-Amur, Russia; 3 N.N. Vorozhtsov Novosibirsk Institute of Organic Chemistry Siberian Branch, Russian Academy of Sciences, Academic Lavrentyev Avenue 9, 630090 Novosibirsk, Russia; 4 Institute of Solid State Chemistry, Siberian Branch, Russian Academy of Sciences, Kutateladze Street 18, 630128 Novosibirsk, Russia; 5 Kurnakov Institute of General and Inorganic Chemistry, Russian Academy of Sciences, Leninskii Avenue 31, 119991 Moscow, Russia

**Keywords:** iron(II) complexes, 2,6-bis(1*H*-imidazol-2-yl)pyridine, X-ray diffraction analysis, EXAFS, Mössbauer, IR, UV-Vis (diffuse reflectance) spectroscopy methods, spin crossover

## Abstract

Novel iron(II) coordination compounds containing a ligand 2,6-bis(1*H*-imidazol-2-yl)pyridine (L), having such a composition as [FeL_2_]SO_4_·0.5H_2_O, [FeL_2_]Br_2_·H_2_O, [FeL_2_](ReO_4_)_2_, [FeL_2_]B_10_H_10_∙H_2_O, [FeL_2_]B_12_H_12_∙1.5H_2_O had been synthesized and studied using UV-Vis (diffuse reflectance), infrared, extended X-ray absorption fine structure (EXAFS), and Mössbauer spectroscopy methods, as well as X-ray diffraction and static magnetic susceptibility methods. The analysis of the μ_eff_(T) dependence in the temperature range of 80–600 K have shown that all the obtained complexes exhibit a high-temperature spin crossover ^1^A_1_ ↔ ^5^T_2_.

## 1. Introduction

Searching for novel coordination compounds wherein the spin state of the central atom can be switched by an external action is an urgent problem. This is indicated by regularly appearing publications in the literature devoted to this topic [1,2,3,4,5,6,7,8]. These compounds include iron(II) complexes with spin crossover (SCO) ^1^A_1_ ↔ ^5^T_2_. The change in spin multiplicity occurs owing to affecting temperature, pressure, irradiation with light of a certain wavelength (LIESST effect), high-frequency magnetic or electric field, and other factors. The transition from a low-spin (LS) to a high-spin (HS) state causes a change in the magnetic, optical, and vibrational properties of the complexes. An important property associated with the spin transition consists in changing metal–donor interatomic distance amounting to 0.2 Å in the case of Fe(II) complexes. Owing to the universal properties of complexes with SCO they have a wide range of potential applications in making optoelectronic, molecular electronic, and spintronic devices [9,10,11,12,13,14,15]. At present, polyfunctional materials combining SCO and other properties are under active study [16,17,18,19].

**Scheme 1:**
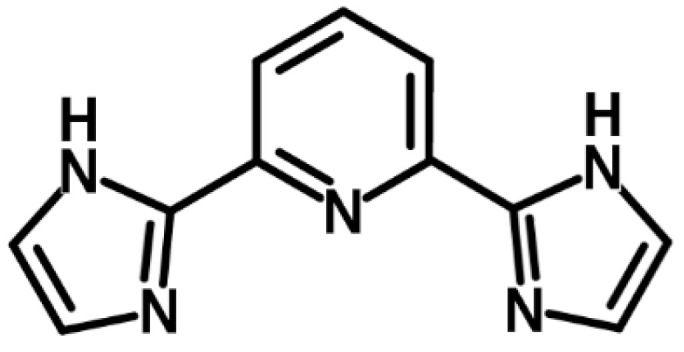
2,6-Bis(1*H*-imidazol-2-yl)pyridine (L).

2,6-Bis(1*H*-imidazol-2-yl)pyridines represent a promising class of potential ligands for the synthesis of complexes with SCO [20]. Earlier, we have reported the studies on iron(II) complexes with 2,6-bis(benzimidazol-2-yl)- and 2,6-bis(4,5-dimethyl-1*H*-imidazol-2-yl)pyridine [21,22,23,24], wherein SCO is exhibited. It seemed worthwhile to continue these investigations into synthesizing and studying Fe(II) complexes with 2,6-bis(1*H*-imidazol-2-yl)pyridine (Scheme 1), which does not contain substituents at the imidazole fragment. Earlier, this ligand was used in order to synthesize complexes with Ru(II), and with a number of metals belonging to the first transition series [25,26,27,28,29]. The X-ray diffraction data have shown that L is coordinated to the metal ion in a tridentate-cyclic manner by two nitrogen atoms belonging to two imidazole rings and one nitrogen atom belonging to pyridine.

## 2. Materials and Methods

### 2.1. Materials

Commercial metal salts and solvents without further purification were used in the synthesis. 2,6-Bis(imidazol-2-yl)pyridine was synthesized as described in [30] (NMR spectra of ligand given in the Supplementary Materials, Figures S1–S4); K_2_B_10_H_10_·2H_2_O, K_2_B_12_H_12_ were obtained according to the procedure [31].

### 2.2. Synthesis of [FeL_2_]SO_4_ 0.5H_2_O (***1·0.5H_2_O***)

A 0.28 g (1 mmol) sample of FeSO_4_·7H_2_O with the addition of 0.1 g of ascorbic acid was dissolved in 10 mL of water under heating; a 0.51 g (2 mmol) L was dissolved in 10 mL of ethanol, and the solutions were then heated and mixed. The resulting solution was evaporated until a red-violet precipitate began to form. After the solution with the precipitate was cooled in a crystallizer with ice, the precipitate was filtered off, washed twice with small portions of water, and dried in air. Yield: 55%. Anal. Calc. for C_22_H_19_FeN_10_O_9/2_S, (583.4): C, 45.3; H, 3.3; N, 24.0. Found: C, 46.2; H, 3.5; N, 23.4.

### 2.3. Synthesis of [FeL_2_]Br_2_·H_2_O (***2·H_2_O***) and [FeL_2_](ReO_4_)_2_ (***3***)

A 0.28 g (1 mmol) sample of FeSO_4_·7H_2_O was dissolved in 5 mL of distilled water acidified with 0.1 g of ascorbic acid. An excess (0.43 g, 1.5 mmol) of KReO_4_ or KBr (0.36 g, 3 mmol) in 10 mL of water and a solution of L (0.51 g, 2 mmol) in 10 mL of ethanol were successively added to the resulting solution under stirring. The resulting solution was evaporated until a red-violet precipitate began to form. After the solution with the precipitate was cooled in a crystallizer with ice, the precipitate was filtered off, washed twice with small portions of water, and dried in air. Yield: 70% (**2·H_2_O**), 30% (**3**). Anal. Calc. for C_22_H_20_Br_2_FeN_10_O, (656.1): C, 40.3; H, 3.1; N, 21.3. Found: C, 41.3; H, 3.2; N, 21.0. Anal. Calc. for C_22_H_18_FeN_10_O_8_Re_2_, (978.7): C, 27.0; H, 1.9; N, 14.3. Found: C, 27.5; H, 2.2; N, 14.6. 

### 2.4. Synthesis of [FeL_2_]B_10_H_10_∙H_2_O (***4∙H_2_O***) and [FeL_2_]B_12_H_12_∙1.5H_2_O (***5·1.5H_2_O***)

A 0.14 g (0.5 mmol) sample of FeSO_4_·7H_2_O was dissolved in 3 mL of distilled water acidified with 0.05 g of ascorbic acid. An excess (0.23 g, 1 mmol) of K_2_B_10_H_10_∙2H_2_O or K_2_B_12_H_12_ (0.22 g, 1 mmol) in 10 mL of water and a solution of L (0.21 g, 1 mmol) in 5 mL of ethanol were added to the resulting solution under stirring. Red-brown precipitates began to form immediately. Each precipitate was filtered off, washed twice with small portions of water and ethanol, and dried in air. Yield: 45% (**4∙H_2_O**), 68% (**5∙1.5H_2_O**). Anal. Calc. for C_22_H_30_B_10_FeN_10_O, (614.5): C, 43.0; H, 4.9; N, 22.8. Found: C, 42.5; H, 5.0; N, 22.1. Anal. Calc. for C_22_H_33_B_12_FeN_10_O_3/2_, (647.1): C, 40.8; H, 5.1; N, 21.6. Found: C, 40.6; H, 5.3; N, 20.9.

### 2.5. Measurement and Characterization

The data for elemental analysis of the complexes was acquired on a EURO EA 3000 analyzer (EuroVector, Pavia, Italy).

**Figure 1:**
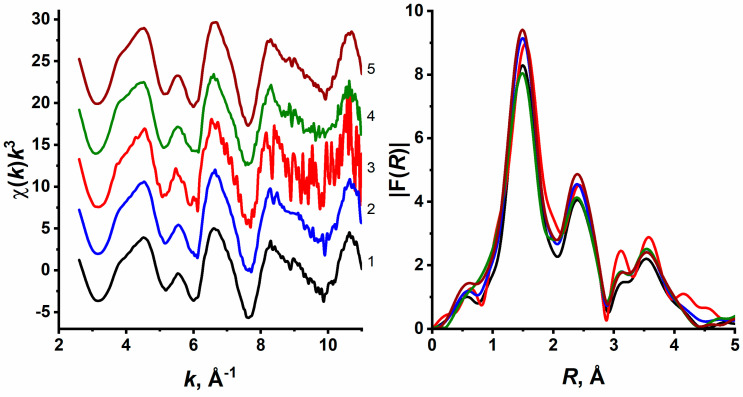
Fe *K–*edge EXAFS spectra (**left**) and their Fourier transform modules without consideration of the phase shift (**right**) of complexes **1**–**5**.

X-ray absorption spectra (XAS) in the Fe K edge region (150 eV before and 800 eV after) were measured in the transmission mode with the use of synchrotron radiation on the 8 beamline, VEPP-3 storage ring at the Siberian Synchrotron and Terahertz Radiation Center (Budker Institute of Nuclear Physics, Siberian Branch, Russian Academy of Sciences, Novosibirsk, Russia) [32]. A Si(111) cut-off crystal was used as a two-crystal monochromator. The operating mode of the storage ring during measurement: energy—2 GeV; current—70–140 mA. For measurements, the samples were mixed with cellulose powder as a filler and pressed into tablets. The mass of the sample was calculated so that the absorption jump at the Fe *K*-edge was 0.8–1. The preprocessing of the absorption spectra (selection of the oscillating part—EXAFS spectra) was performed with the use of the VIPER 10.17 program [33]. The “radial pair distribution function” (Figure 1) was obtained by the Fourier transform of the *k*^3^-weighted EXAFS function in the range of wave vectors *k* = 2.0–11.0 Å^−1^.

The local environment of the Fe ion [interatomic distances (*R*_i_) and angles (*Q*_i_), coordination numbers (*N*_i_), and Debye–Waller factors (*σ*^2^)] was modeled using the EXCURVE 98 cod [34]. In this program phase and amplitude characteristics were calculated in the von-Bart and Hedin approximation. The amplitude suppression factor S_0_^2^ due to multielectron processes was determined for the crystallographically characterized compound (S_0_^2^ = 0.85) and fixed during further modeling of the studied compounds spectra. The Debye–Waller factor was the same separately for nitrogen and carbon atoms.

IR spectra were taken on a Scimitar FTS 2000 in the range of 4000–400 cm^−1^ and a Vertex 80 in the range of 600–100 cm^−1^. Compound samples were prepared as suspensions in vaseline and fluorinated oils and in polyethylene.

The Kubelka-Munk diffuse reflectance spectra were obtained on a Shimadzu UV-3101 PC scanning spectrometer.

The XRD investigation of polycrystalline samples was performed using a Shimadzu XRD 7000 diffractometer (CuK_α_ radiation).

The static magnetic susceptibility was measured using Faraday balance type setup equipped with electromagnetic compensating torsion quartz microbalance. The Delta DTB9696 temperature controller (Delta Electronics Inc., Taipei, Taiwan) was used for the investigated compounds temperature stabilization (~1 K) in the range of temperatures 80–600 K. The temperature scanning rate for the process of heating or cooling samples was ~1 K/min. The magnetic field strength (7300 Oe) stabilization precision was ~2%. The compounds studied were sealed in quartz cellules filled with atmospheric air at 760 Torr. In order to study the effect of crystallization water, the samples were placed in open quartz ampoules and vacuumed at 10^−2^ Torr, after which the helium atmosphere at 5 Torr was formed.

The effective magnetic moment of studied compounds was calculated as:

μeff=(3kNAμB2⋅χT)12≈(8χT)12,

*k*—Boltzmann constant, *N_A_*—Avogadro constant, and *µ_B_*—Bohr magneton. In the above formula, the diamagnetic contribution in total magnetic susceptibility (*χ*) was taken into account using the method of Pascal’s constants. The direct (*T*_c_↑) and reverse (*T*_c_↓) transitions temperatures were obtained using condition the magnetic moment second derivative zero value (*d^2^*(*µ_eff_*(*T*))/*dT*^2^ = 0).

The Mössbauer spectra were collected with a spectrometer NP-610 (KFKI, Budapest, Hungary), using ^57^Co in a metal Rh matrix as a radioactive source. The spectra were measured at a room temperature. The spectra were processed to find the values of isomer shift δ and quadrupole splitting ε. The isomer shifts are given relative to metal iron.

## 3. Results and Discussion

Iron(II) complexes with 2,6-bis(1*H*-imidazol-2-yl)pyridine were isolated from aqueous-ethanol solutions. To maintain the oxidation state of iron(II), ascorbic acid was added to the solution.

Complex **1·0.5H_2_O** was synthesized by reacting stoichiometric amounts of FeSO_4_ and L. Syntheses **2·H_2_O**, **3**, **4∙H_2_O**, **5·1.5H_2_O** were carried out in two stages. At the first stage, the corresponding iron(II) salts were obtained by adding a 1.5- or 2-fold excess of KBr, KReO_4_, K_2_B_10_H_10_ or K_2_B_12_H_12_ into a solution of FeSO_4_ salt. At the second stage, a solution of the ligand in ethanol was added to the resulting salt solution. The obtained diffraction patterns show that the synthesized compounds are crystalline, while [FeL_2_]SO_4_·0.5H_2_O and [FeL_2_]Br_2_·H_2_O, as well as [FeL_2_]B_10_H_10_∙H_2_O and [FeL_2_]B_12_H_12_∙1.5H_2_O are isotypical.

**Figure 2:**
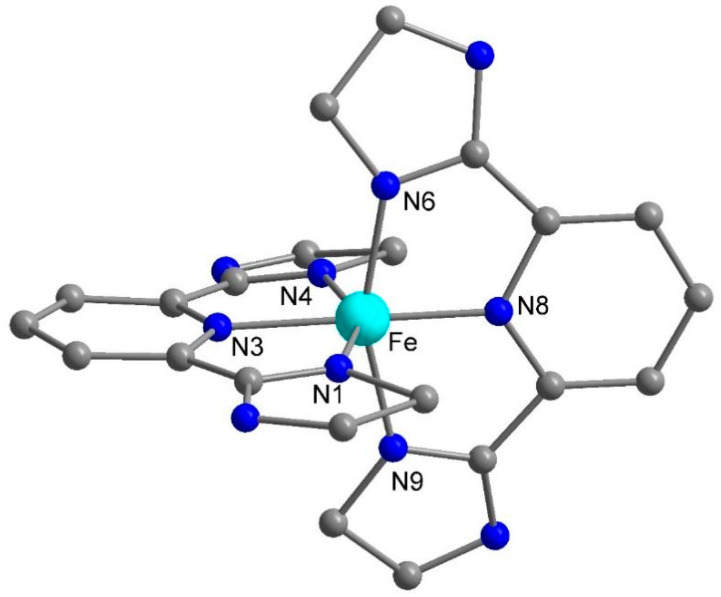
General view of the structure of the coordination core obtained by means of EXAFS spectra simulation for such complexes as **1·0.5H_2_O**, **2·H_2_O**, **4∙H_2_O**, **5·1.5H_2_O**.

The structure of the whole molecule for all complexes in the LS state (T = 297 °C) was established from the EXAFS data using the multiple scattering approximation within the software package EXCURV (except for [FeL_2_](ReO_4_)_2_ (**3**)) excluding hydrogen atoms, and anions that exert only a weak effect on the shape of the EXAFS spectrum owing to their spatial separation from the central iron ion. For complex **3**, the signal-to-noise ratio in the EXAFS spectrum is worse than that for the spectra of other complexes owing to the presence of a heavy anion ReO_4_^−^. Simulation of the whole molecule in the multiple scattering approximation gives too large errors in determining the parameters. Therefore, the simulation of the EXAFS spectrum for complex **3** could be carried out only in a single scattering approximation. The structure of the complexes in the LS state (by the example of complex **4∙H_2_O**) obtained by means of EXAFS spectra simulation is presented in Figure 2.

Table 1 lists the microstructure parameters of the coordination site for complexes **1·0.5H_2_O**, **2·H_2_O**, **4∙H_2_O**, **5·1.5H_2_O**.

The structure of the coordination site of complex [FeL_2_](ReO_4_)_2_ (**3**) in the LS state was obtained from modeling the spectrum filtered in real space (Δ*R* = 0.9 to 3.0 Å). The simulation data are presented in Table 2. The coordination numbers of the nearest spheres of the iron ion environment were fixed in accordance with the data obtained in the simulation of complexes in the LS state in the multiple scattering approximation.

**Figure 3:**
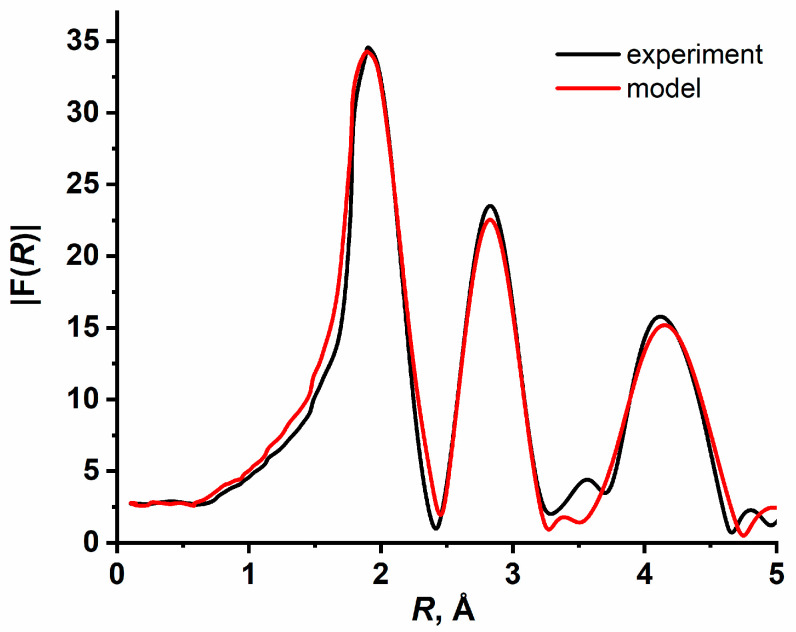
Comparison of the experimental and model radial distribution function for complex [FeL_2_]B_10_H_10_∙H_2_O.

Figure 3 illustrates a comparison of the experimental and simulated radial distribution function for [FeL_2_]B_10_H_10_∙H_2_O. The simulation was carried out using a multiple scattering approximation.

Table 3 and Figures S5–S7 show the main vibration frequencies in the spectra of L and Fe(II) complexes. In the high-frequency spectral region for **1·0.5H_2_O**, **2·H_2_O**, **4∙H_2_O**, **5·1.5H_2_O,** ν(OH) vibrations are observed; in the wave number range of 3200–3050 cm^−1^ for all the complexes there are stretching vibrations of NH-groups, and in the range of 3100–2850 cm^–1^ there are vibrations of ν(CH). In the wave number range of 1650–1450 cm^−1^, there are stretching and bending vibration bands inherent in heterocyclic rings. The spectra of the complexes in the range of ring vibrations exhibit a change in the number and position of the imidazole and pyridine bands in comparison with the spectrum of the ligand, which indicates the coordination of nitrogen atoms of the rings to Fe(II) ions. The presence of non-split bands inherent in stretching vibrations characteristic of the SO_4_^2−^ and ReO_4_^−^ anions indicates the fact that they are in outer-sphere position. The vibration bands of the B–H bonds of the outer-sphere anions B_10_H_10_^2−^ and B_12_H_12_^2−^ are centered at the wave numbers of 2470 (ν(BH)) and 1075 cm^−1^ (δ(BBH)). In the case of the spectra for **4∙H_2_O**, **5·1.5H_2_O** they are shifted with respect to those observed in the spectra of the initial salts, which could be, to all appearance, caused by the formation of H_2_O^δ− … δ+^H–B bonds. In the far region of the spectra of all the complexes, Fe(3d^6^)–ligand(π) charge-transfer transition bands and vibration bands (M-N) are observed. The position of these bands is typical for the spectra of low-spin octahedral iron(II) complexes [35].

The diffuse reflectance spectra (DRS, Figures S8–S12) of all the obtained complexes exhibit intense metal-ligand charge transfer bands ν_1_(*e_g_* → $\pi_{L}^{*}$) in the wavelength range of 300–350 nm (λ_max_ ≈ 324–326 nm). The DRS of **4∙H_2_O** and **5∙1.5H_2_O** exhibit three absorption bands, too, (see Table 4); they correspond to the ^1^A_1_ → ^1^T_2_, ^1^A_1_ → ^1^T_1_ and ^1^A_1_ → ^1^A_2_ transitions in the strong octahedral field of the ligands. For low-spin axially distorted octahedral iron(II) complexes, the term ^1^T_1_ is transformed according to the representation of ^1^E + ^1^A_2_, whereas term ^1^T_2_ is transformed according to the representation of ^1^E + ^1^B_2_ [36]. Therefore, the ^1^A_1_ → ^1^B_2_, ^1^A_1_ → ^1^A_2_ and ^1^A_1_ → ^1^E wide transition bands (see Table 4) observed in the spectra of **1·0.5H_2_O**, **2·H_2_O** and **3**, indicate the fact that an axial distortion of the octahedral coordination of these complexes occur. Low-intensity transitions and overlapping three wide bands do not make it possible to perform reliable quantitative calculations of crystal field parameters in this case. The spectra of all the complexes do not exhibit the ^5^T_2_ → ^5^E band, which is caused by the HS state of iron(II). We were able to calculate the splitting parameters based on the difference between ^1^A_1_ → ^1^T_2_ and ^1^A_1_ → ^1^T_1_ absorption frequencies [36] for low-spin (LS) forms of complexes with *closo*-borate anions. The B values were computed using the formula 16B = [ν (^1^A_1_ → ^1^T_2_) − ν (^1^A_1_ → ^1^T_1_)]. The values C and Δ_LS_ (Table 4) were calculated using the equations: ν_LS_ = Δ_LS_ − C + 86B^2^/Δ_LS_ and C = 4.41·B [36,37,38]. The obtained data show that 2,6-bis(1*H*-imidazol-2-yl)pyridine is a strong field ligand. In addition, these data, as well as the values obtained for a number of previously synthesized Fe(II) complexes with 2,6-bis(benzimidazol-2-yl)pyridine and 2,6-bis(4,5-dimethyl-1*H*-imidazole) [22,23,24], obey the inequality that reflects the condition for the manifestation of SCO [37]: 19.000 cm^−1^ ≤ Δ_LS_ ≤ 22.000 cm^−1^. 

**Figure 4:**
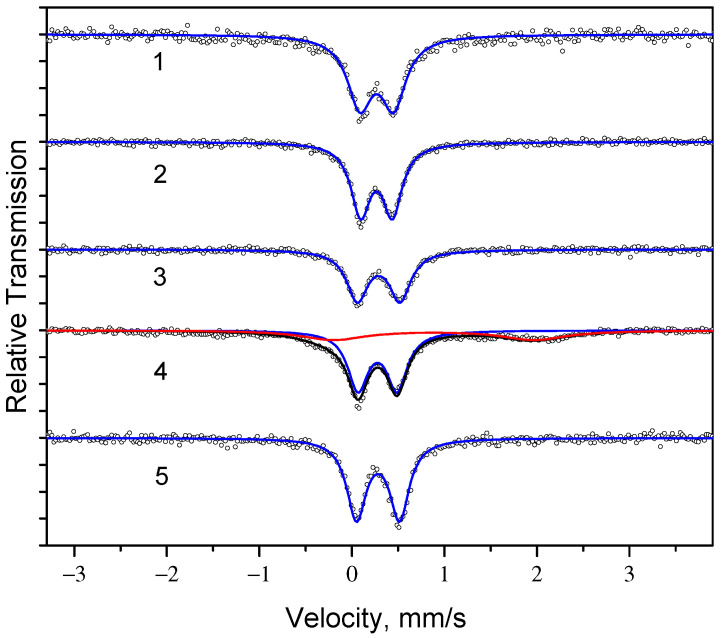
Mössbauer spectra of complexes **1·0.5H_2_O**, **2·H_2_O**, **3**, **4∙H_2_O**, **5·1.5H_2_O** (1–5).

The Mössbauer spectra of complexes **1·0.5H_2_O**, **2·H_2_O**, **3** and **5·1.5H_2_O** represent quadrupole doublets whose parameters correspond to the LS state of iron(II) (Figure 4). The spectrum of **4·H_2_O** also exhibits a broadened doublet related to the HS form of the complex (36%). The parameters of the Mössbauer spectra are presented in Table 5. 

**Figure 5:**
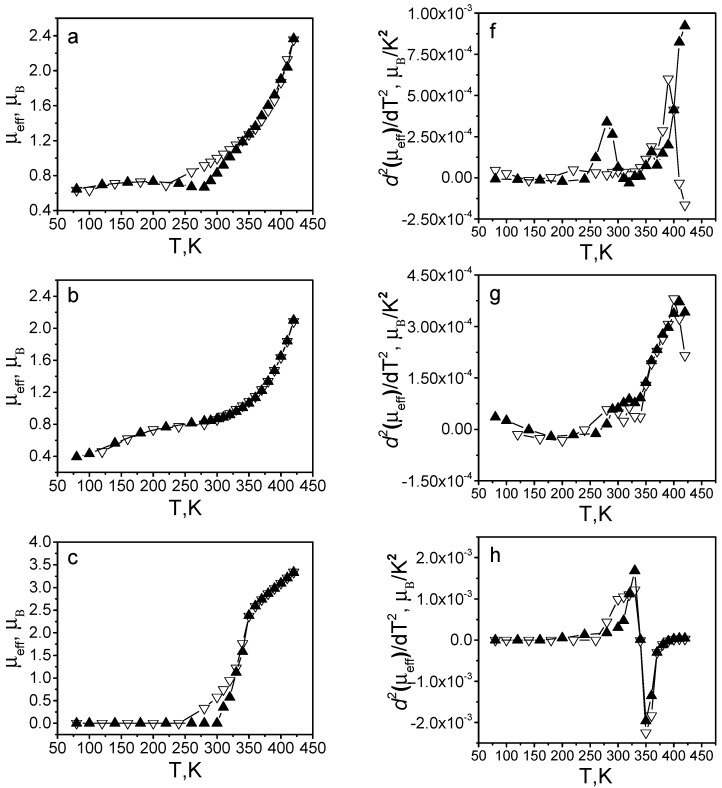

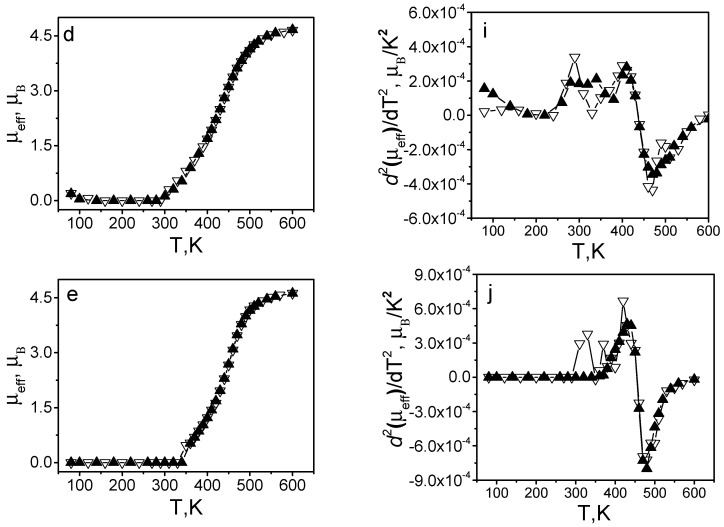
Dependences of µ_eff_(T) (**a**–**e**) and d^2^(µ_eff_(T))/dT^2^ (**f**–**j**) for **1·0.5H_2_O** (**a**,**f**), **2·H_2_O** (**b**,**g**), **3** (**c**,**h**), **4·H_2_O** (**d**,**i**), **5·1.5H_2_O** (**e**,**j**). The white and black triangles in the figure correspond to the samples cooling and heating processes respectively.

The temperature dependences of the effective magnetic moment for the analyzed complexes are shown in Figure 5. Spin crossover (SCO) is observed for all the compounds under investigation. In the case of complexes **1·0.5H_2_O, 2·H_2_O, 3** the µ_eff_ values observed in the thermal stability range (2.35, 2.1 and 3.35 µ_Β_, respectively), are significantly lower than the theoretical spin-only value of 4.9 µ_Β_ for the Fe(II) ion. The **4∙H_2_O** and **5∙1.5H_2_O** complexes are more stable and thus a temperature of 600 K could be reached. For these compounds, a complete spin-crossover is observed. However, the µ_eff_ values observed in the HS state of these compounds are also lower than the theoretical value for Fe(II). It should be noted that the experimental µ_eff_ values for **4∙H_2_O** and **5∙1.5H_2_O** complexes are in the range of 4.6–5.7 observed for Fe(II) compounds [39,40]. In the case of **1·0.5H_2_O** and **2·H_2_O** the residual effective magnetic moment in LS state (0.65 and 0.4 µ_Β_, respectively), is presented by µ_eff_(T) dependences. This fact could be connected with temperature-independent paramagnetism. Complexes **3**_,_ **4∙H_2_O** and **5∙1.5H_2_O** in LS state exhibit diamagnetism with a zero µ_eff_ value. Despite the fact that low µ_eff_ values are achieved in the investigated temperature range for **1·0.5H_2_O** and **3**, the condition of d^2^(µ_eff_(T))/dT^2^ = 0 could be satisfied and some values of SCO temperature could be determined. Table 6 shows the temperature values of direct (T_c_↑) and inverse (T_c_↓) transitions. For the [FeL_2_]Br_2_·H_2_O one could suggest that the SCO temperature is higher than 420 K. The determined temperature of the inverse transition increases in a series of [FeL_2_](ReO_4_)_2_ → [FeL_2_]SO_4_·0.5H_2_O → FeL_2_]B_10_H_10_·H_2_O → [FeL_2_]B_12_H_12_·1.5H_2_O.

**Figure 6:**
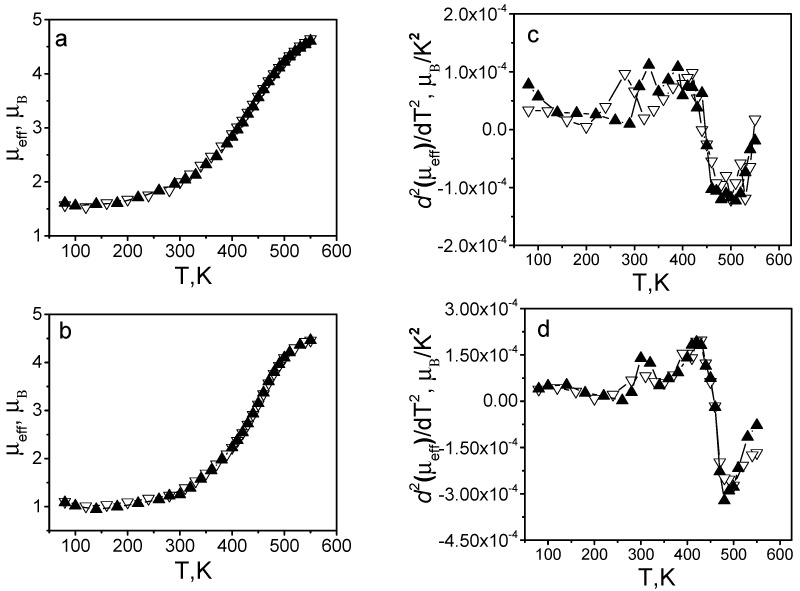
Dependences of µ_eff_(T) (**a**,**b**) and d^2^(µ_eff_(T))/dT^2^ (**c**,**d**) for dehydrated complexes **4** (**a**,**c**), **5** (**b**,**d**). The white and black triangles in the figure correspond to the samples cooling and heating processes respectively.

The effect of the crystallization of water has been studied for **4·H_2_O** and **5·1.5H_2_O** complexes (Figure 6). It should be noted that in the case of rarefied atmosphere the decomposition of the dehydrated complexes occurs in a lower temperature range than it is observed for initial compounds. Nevertheless, the SCO has been observed in this case, too. The µ_eff_ value of 4.65 µ_Β_ achieved in HS state for **4** corresponds to the value observed for the initial complex. In the case of **5** complex, the µ_eff_ value (4.6 µ_Β_) exhibits an increase after dehydration. Residual µ_eff_ values (~1–1.5 µ_Β_) have been registered to occur for both complexes in the LS state. The SCO temperature increases after dehydration; however, the **5** complex demonstrates the highest SCO temperature values as it is observed in the case of the initial compound. Thus, the dehydration of **4·H_2_O** and **5·1.5H_2_O** complexes lead to appearing residual µ_eff_ value and to an increase in the temperature of SCO.

## 4. Conclusions

In this work, we have synthesized and investigated five novel coordination compounds of various iron(II) salts with 2,6-bis(1*H*-imidazol-2-yl)pyridine (L). The structure of the coordination core of the complexes has been determined by means of EXAFS spectra simulation. Two ligand molecules are coordinated to the iron(II) ion in a tridentate-cyclic fashion by the nitrogen atom belonging to pyridine and two nitrogen atoms belonging to imidazole rings. Thus, the complexes have the distorted-octahedral structure of the coordination polyhedron, the FeN_6_ core. The studies on the µ_eff_(T) dependence have shown that complexes having such a composition as [FeL_2_]SO_4_·0.5H_2_O, [FeL_2_]Br_2_·H_2_O, [FeL_2_](ReO_4_)_2_, [FeL_2_]B_10_H_10_·H_2_O, [FeL_2_]B_12_H_12_·1.5H_2_O exhibit an ^1^A_1_ ↔ ^5^T_2_ high-temperature spin crossover. A comparison of the data obtained for the synthesized compounds with those obtained by us earlier [21,22,23,24] shows that spin-crossover ^1^A_1_ ↔ ^5^T_2_ is observed in all complexes of Fe(II) with 2,6-bis(imidazole-2-yl)pyridines. The temperatures of direct SCO, T_c_↑, in most compounds are significantly higher than room temperature.

## Figures and Tables

**Table 1:** Microstructure parameters of the Fe coordination site for the complexes at T = 300 K (LS) obtained by EXAFS fitting with multiple scattering approximation (*R*_i_—interatomic distance, 2*σ*_i_^2^—Debye–Waller factor, *F*_i_—the statistical error of the fitting).

Bonds	*R*_i_, Å				Angles	Ω, Deg.			
	1·0.5H_2_O	2·H_2_O	4∙H_2_O	5·1.5H_2_O		1·0.5H_2_O	2·H_2_O	4∙H_2_O	5·1.5H_2_O
Fe(1)-N(1)	1.96	1.97	1.98	1.94	N(1)Fe(1)N(8)	107.9	102.6	105.9	102.6
Fe(1)-N(3)	1.96	1.91	1.93	1.95	N(1)Fe(1)N(6)	95.3	92.5	95.1	100.1
Fe(1)-N(4)	1.95	1.97	1.98	1.93	N(1)Fe(1)N(9)	97.1	92.1	96.6	102.2
Fe(1)-N(6)	1.95	1.96	1.98	1.96	N(1)Fe(1)N(3)	75.2	79.8	75.6	85.3
Fe(1)-N(8)	1.86	1.88	1.90	1.86	N(3)Fe(1)N(9)	97.8	102.1	100.3	102.9
Fe(1)-N(9)	1.96	1.95	1.96	1.96	N(3)Fe(1)N(4)	75.8	81.2	80.6	73.7
2*σ*^2^ (Fe-N), Å^2^	0.014	0.013	0.013	0.012	*F*_i_ (*)	2.7	2.5	1.8	1.6

* The determination accuracy for parameters (interatomic distances and angles) based on the EXAFS data is ±1% (for the nearest sphere of the environment). $F_{i}=\sum_{i}^{N}w_{i}^{2}{\left(\chi_{i}^{exp}\left(k\right)-\chi_{i}^{th}\left(k\right)\right)}^{2},w_{i}=\frac{k_{i}^{n}}{\sum_{i}^{N}k_{i}^{n}\vee \chi_{j}^{exp}\left(k\right)\vee }$.

**Table 2:** Microstructure parameters for complex [FeL_2_](ReO_4_)_2_ in the LS state obtained by single-scattering EXAFS data fitting.

Compound	Central Ion–Scattering Atom	*N*_i_	*R*_i_, Å	2*σ*_i_^2^, Å^2^	*F*_i_
[FeL_2_](ReO_4_)_2_	Fe–N	6	1.96	0.010	2.7
	Fe–C	8	2.78	0.014	
	Fe–C	4	3.20		

**Table 3:** The main vibrational frequencies (cm^−1^) in the spectra of L and complexes.

L	1·0.5H_2_O	2·H_2_O	3	4·H_2_O	5·1.5H_2_O	Assignment
	3485	3433		3225	3269	*ν*(OH)
3100_w_	3020_w_	3147 3116	3050_w_	3151 3130	3148 3129	*ν*(NH)
3079 3035	3060 3021	3067	3067	2954 2924 2854	2988 2040 2849	*ν*(CH)
1598 1573 1552	1595 1570 1550	1569 1551 1479	1593 1566 1552	1622 1558 1490 1469	1622 1558 1490 1471	R_ring_
	1118 1085 1070					ν(SO_4_)
			903			ν(ReO_4_)
				2473	2493	ν(B-H)
				1086 1036	1105 1054	δ(BBH)
	496	491	485	485	484	Fe(3d^6^)–ligand (π) charge-transfer transition
	385 356 330	376 334	381 321	334	333	*ν*(M-N)

**Table 4:** Parameters of diffuse reflectance spectra.

Complex	λ(^1^A_1_ → ^1^T_2_)	λ(^1^A_1_ → ^1^B_2_)	λ(^1^A_1_ → ^1^T_1_)	λ(^1^A_1_ → ^1^A_2_)	λ(^1^A_1_ → ^1^E)	Calculated Parameter		
						B	C	∆_LS_
**1·0.5H_2_O**		454		613	536			
**2·H_2_O**		454		624	539			
**3**		432		603	503			
**4·H_2_O**	475		518	620		109.3	482.0	1.97 × 10^4^
**5·1.5H_2_O**	465		518	620		137.5	606.5	1.98 × 10^4^

**Table 5:** Mössbauer spectra parameters of the complexes.

Complex	δ, mm/s	ε, mm/s	Γ, mm/s
**1·0.5H_2_O**	0.269	0.364	0.33
**2·H_2_O**	0.266	0.342	0.25
**3**	0.288	0.464	0.28
**4∙H_2_O**	0.278 (64%) 0.925 (36%)	0.420 2.224	0.26 0.80
**5·1.5H_2_O**	0.282	0.465	0.25

**Table 6:** The temperatures of direct (T_c_↑) and inverse (T_c_↓) SCO for the studied complexes.

Complex	T_c_↑, K	T_c_↓, K
[FeL_2_]SO_4_·0.5H_2_O	>420	409
[FeL_2_]Br_2_·H_2_O	>420	>420
[FeL_2_](ReO_4_)_2_	340	340
[FeL_2_]B_10_H_10_·H_2_O	436	436
[FeL_2_]B_12_H_12_·1.5H_2_O	455	455
[FeL_2_]B_10_H_10_	447	440
[FeL_2_]B_12_H_12_	458	458

## Data Availability

The data presented in this study are available on request from the corresponding authors.

## Supplementary Materials

The following supporting information can be downloaded at: https://www.mdpi.com/article/10.3390/molecules27165093/s1, Figure S1: NMR ^1^H of 2,6-di(1*H*-imidazol-2-yl)pyridine, 100 MHz, NS 24; Figure S2: NMR ^13^C of 2,6-di(1*H*-imidazol-2-yl)pyridine, 100 MHz, NS 96; Figure S3: NMR ^13^C of 2,6-di(1*H*-imidazol-2-yl)pyridine, 100 MHz, NS 7376; Figure S4: NMR ^13^C of 2,6-di(1*H*-imidazol-2-yl)pyridine, 100 MHz, NS 176; Figure S5: IR spectrum of 2,6-bis(1*H*-imidazol-2-yl)pyridine (L); Figure S6: IR spectrum of [FeL_2_]B_10_H_10_∙H_2_O (4∙H_2_O); Figure S7: IR spectrum of [FeL_2_]B_12_H_12_∙1.5H_2_O (5·1.5H_2_O); Figure S8: Comparison of the experimental DRS and model for complex [FeL_2_]SO_4_∙0.5H_2_O; Figure S9: Comparison of the experimental DRS and model for complex [FeL_2_]Br_2_∙H_2_O; Figure S10: Comparison of the experimental DRS and model for complex [FeL_2_](ReO_4_)_2_; Figure S11: DRS for complex [FeL_2_]B_10_H_10_∙H_2_O; Figure S12: DRS for complex [FeL_2_]B_12_H_12_∙1.5H_2_O.

## Sample Availability

Samples of the compounds are available from the authors on request.
