# Supplementary material for: High-Temperature Spin Crossover in Iron(II) Complexes with 2,6-Bis(1H-imidazol-2-yl)pyridine
Source: Molecules. 2022 Aug 10;27(16):5093. doi: 10.3390/molecules27165093 (PMC9415506; doi:10.3390/molecules27165093)
Supplement: Supplementary file 1 [file molecules-27-05093-s001.zip › molecules-1786821-supplementary.pdf]

Supplementary Materials

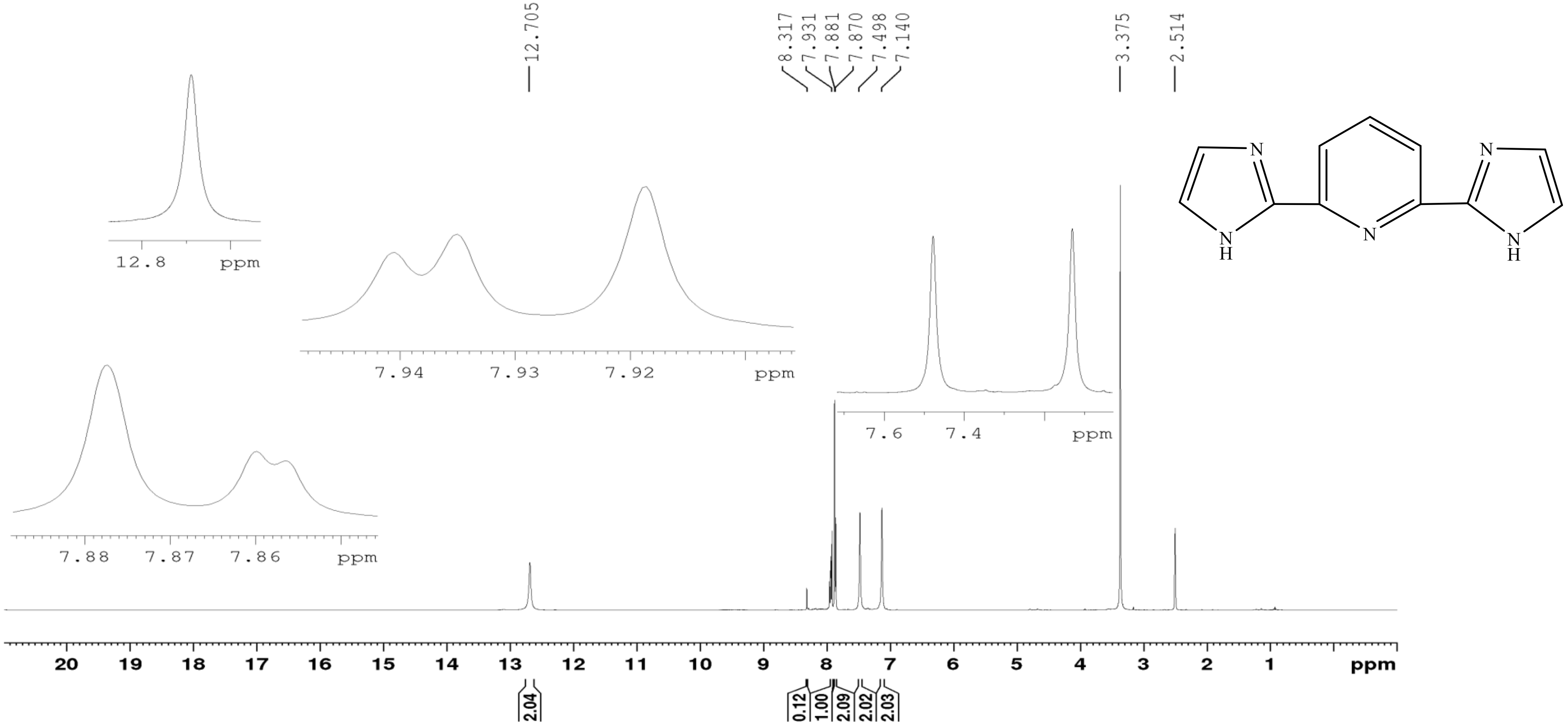

Figure S1. NMR ^{1}H of 2,6-di(1*H*-imidazol-2-yl)pyridine, 100 MHz, NS 24.

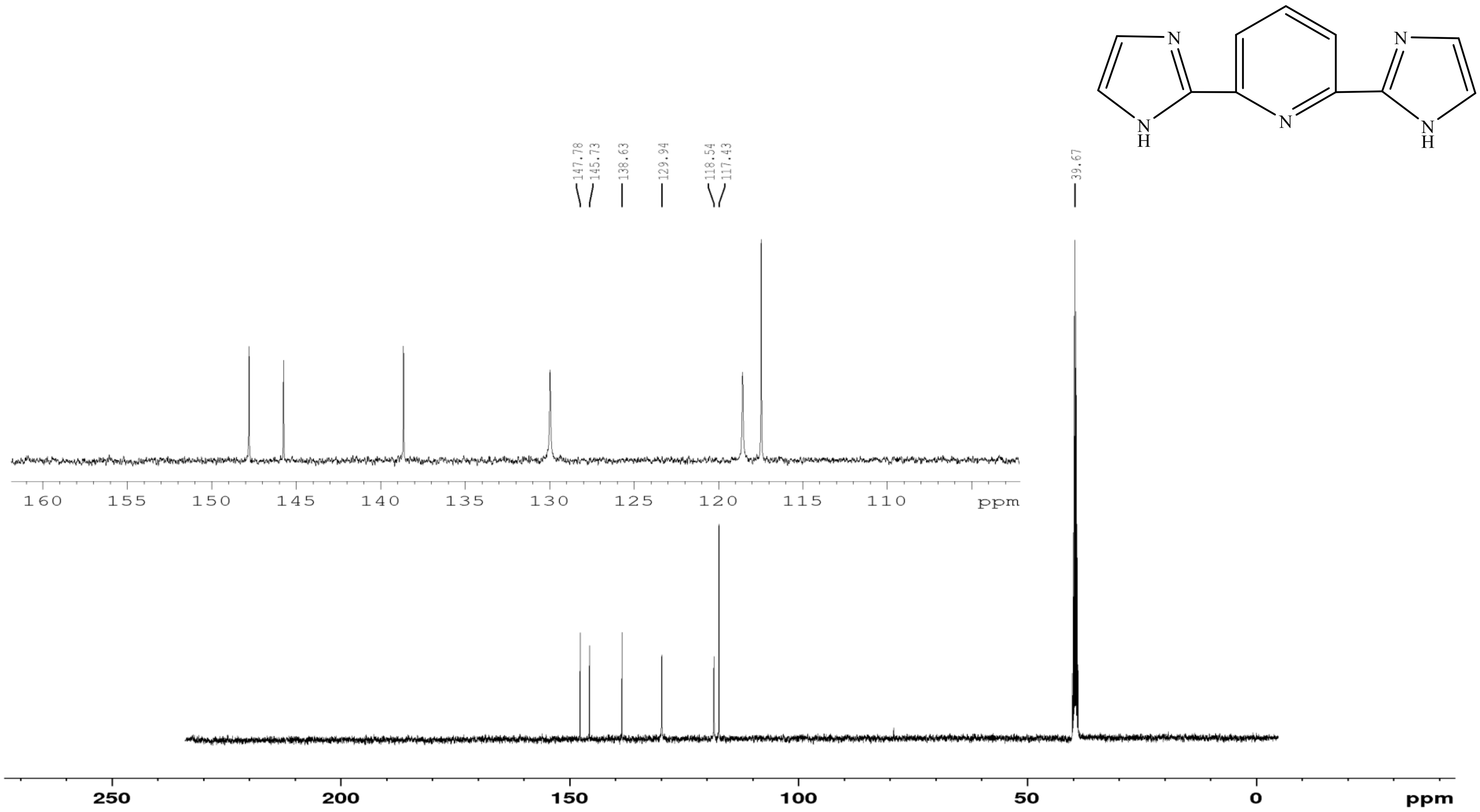

Figure S2. NMR ^{13}C of 2,6-di(1*H*-imidazol-2-yl)pyridine, 100 MHz, NS 96.

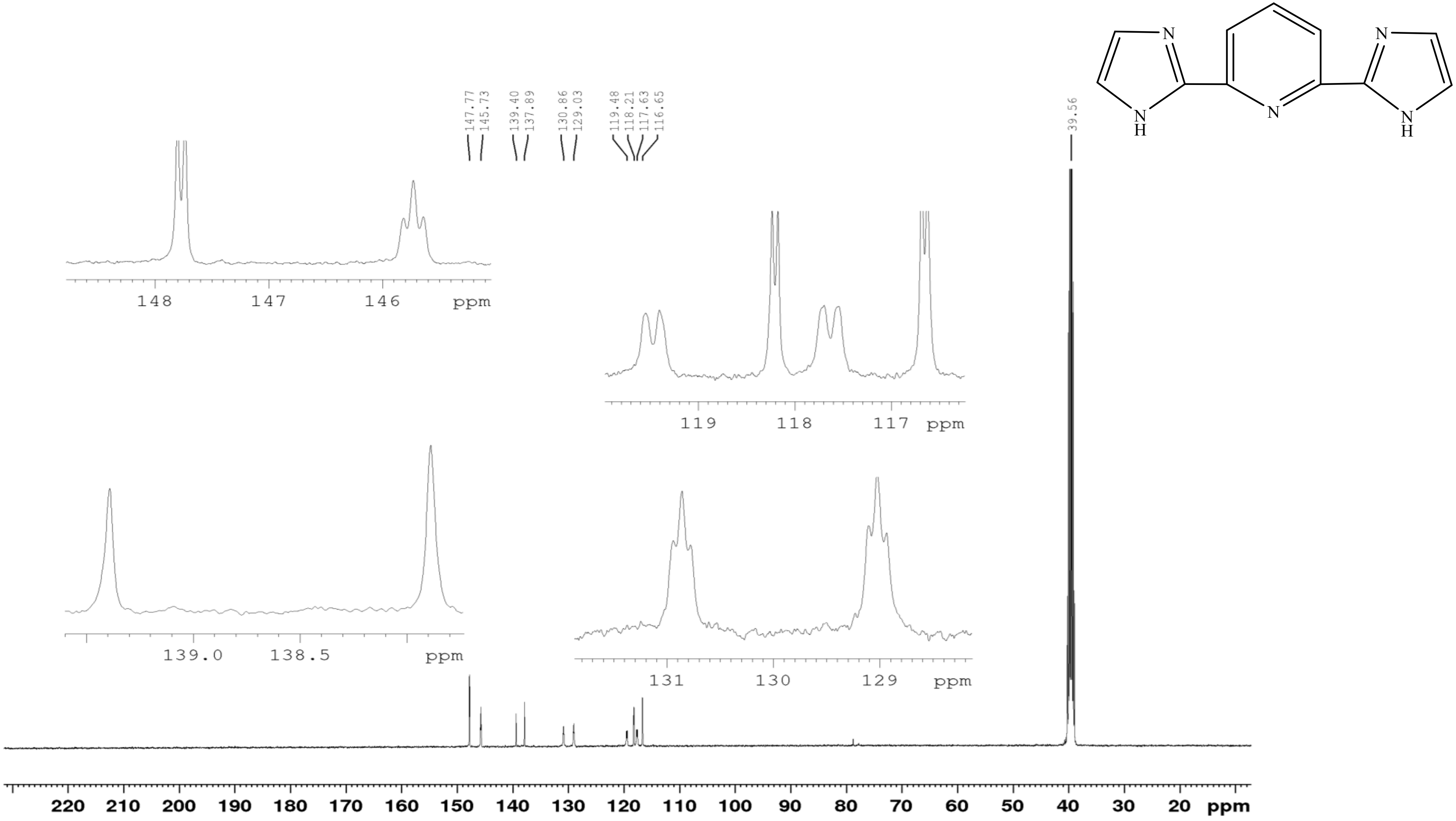

Figure S3. NMR ^{13}C of 2,6-di(1*H*-imidazol-2-yl)pyridine, 100 MHz, NS 7376.

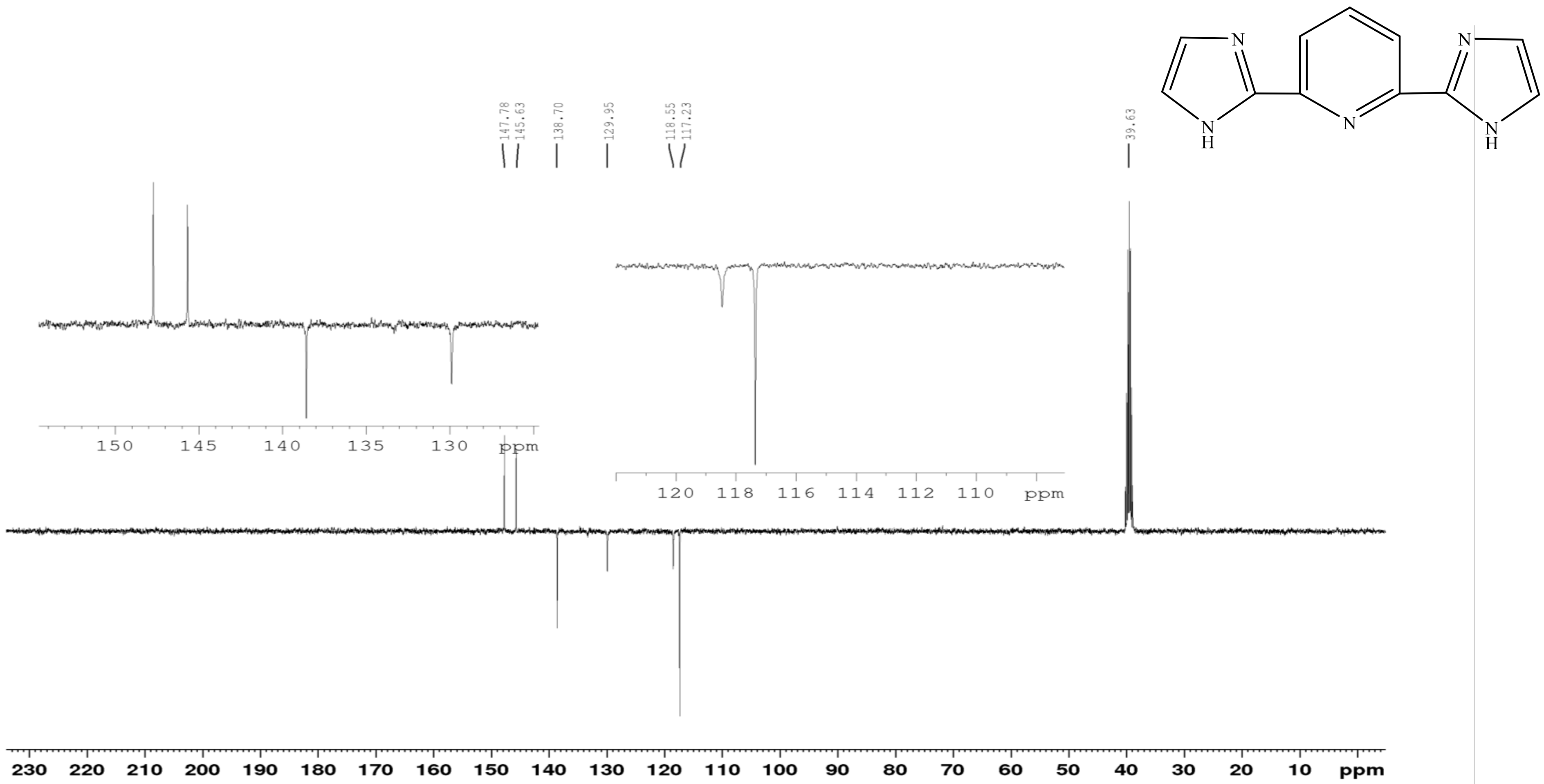

Figure S4. NMR ^{13}C of 2,6-di(1*H*-imidazol-2-yl)pyridine, 100 MHz, NS 176.

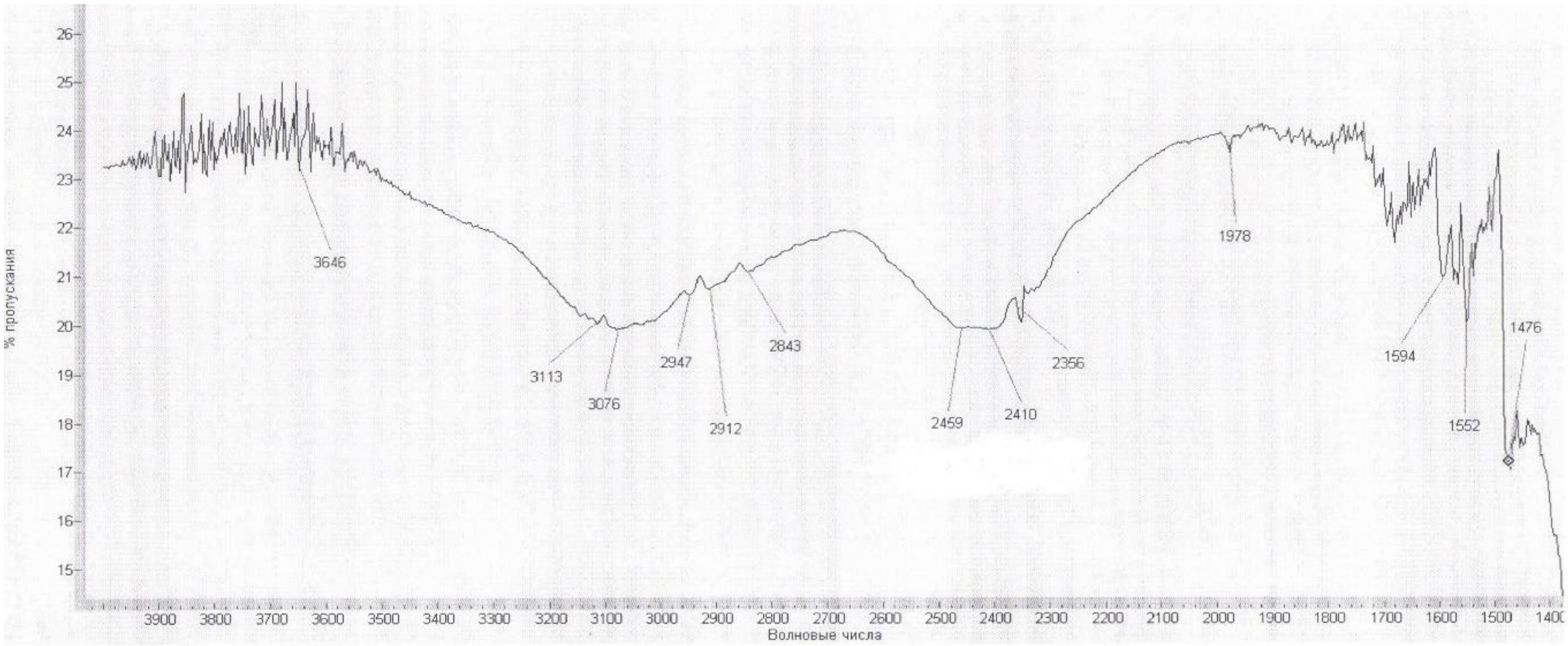

Figure S5. IR spectrum of 2,6-bis(1H-imidazol-2-yl)pyridine (L).

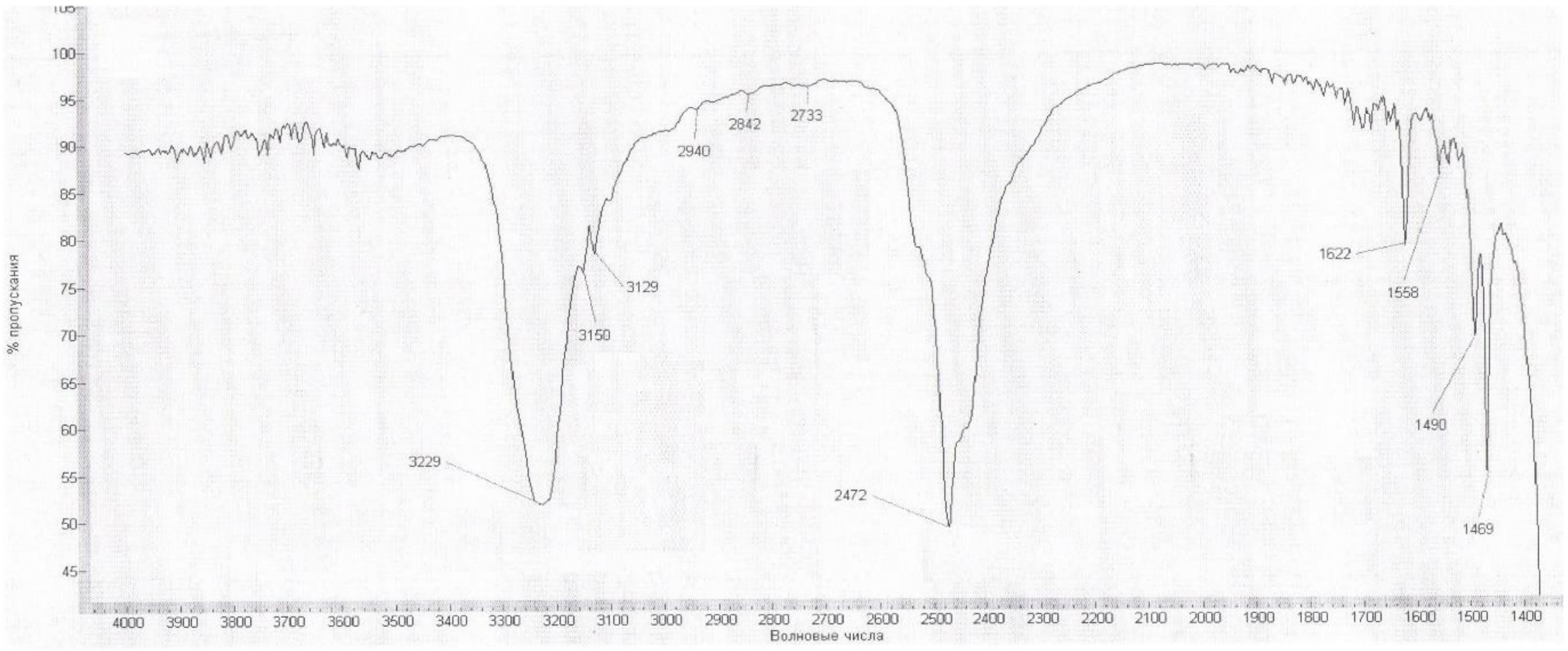

Figure S6. IR spectrum of $[FeL_2]B_{10}H_{10}\cdot H_2O$ ($4\cdot H_2O$).

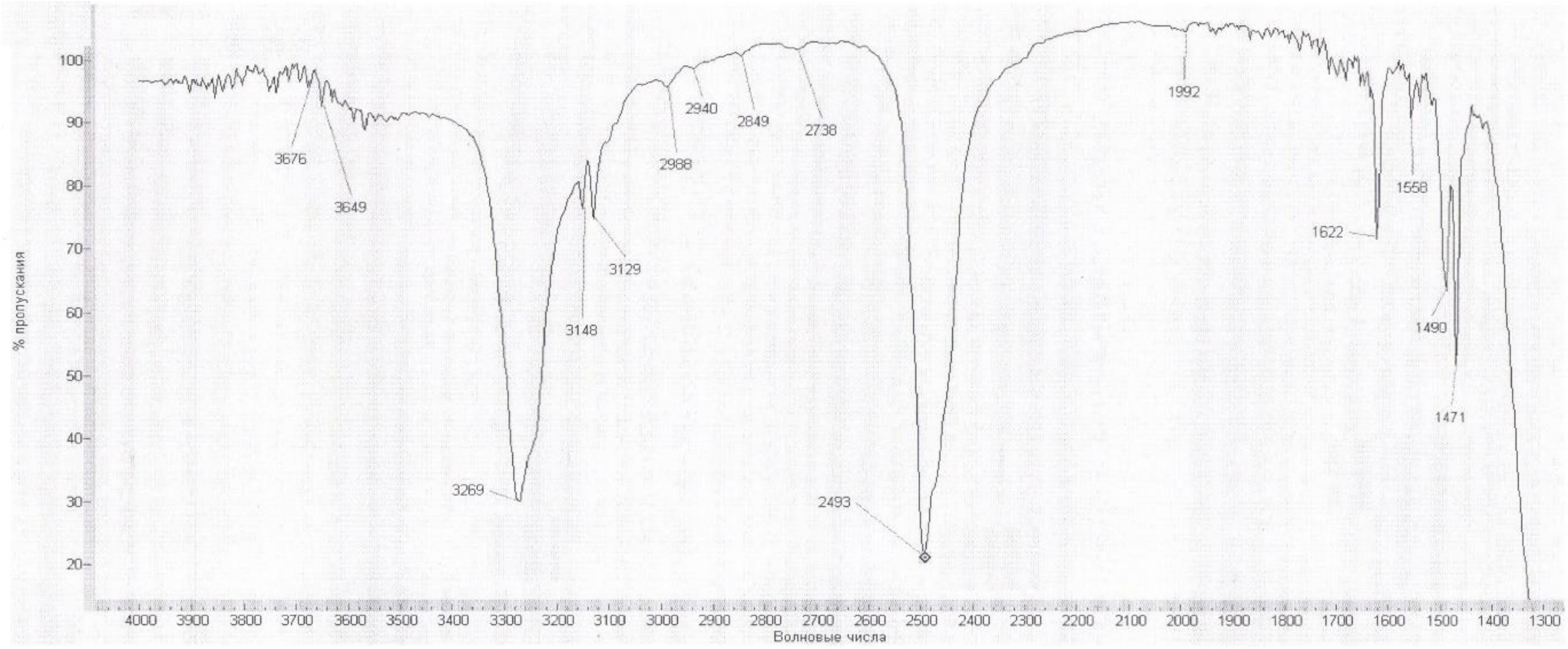

Figure S7. IR spectrum of $[FeL_2]B_{12}H_{12}\cdot 1.5H_2O$ ($5\cdot 1.5H_2O$).

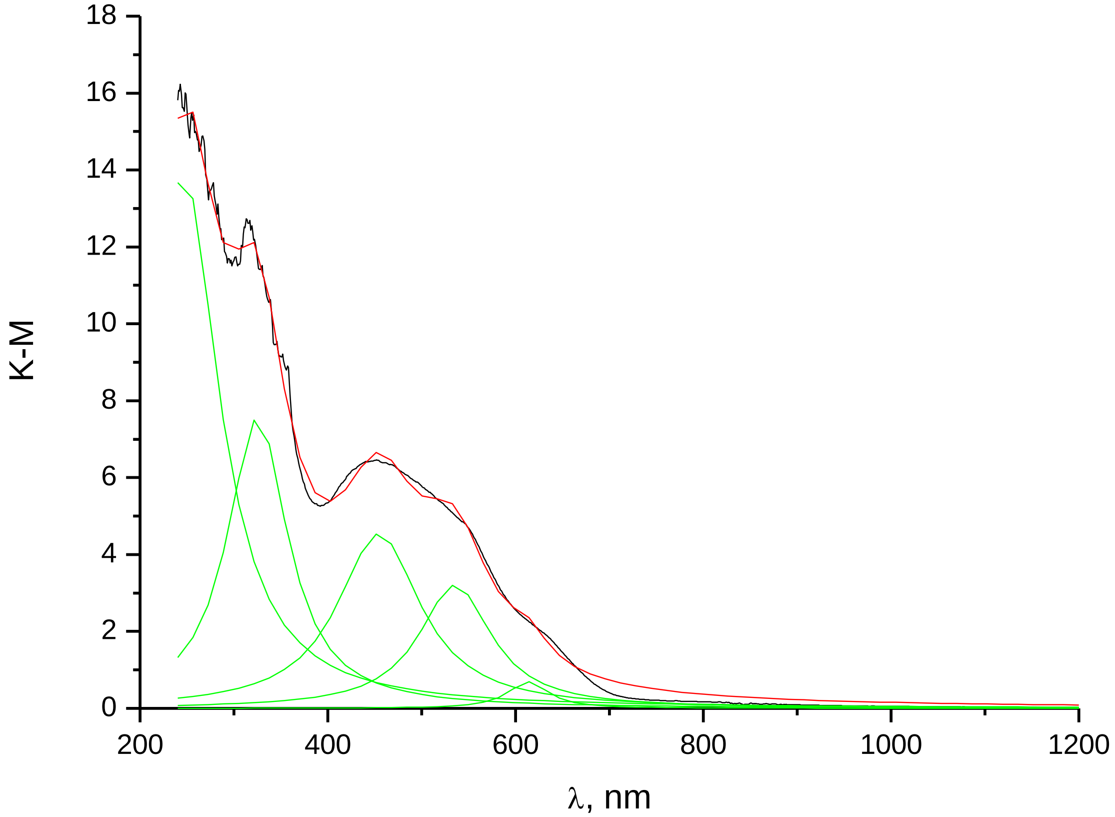

Figure S8. Comparison of the experimental DRS and model for complex $[FeL_2]SO_4{\cdot}0.5H_2O$.

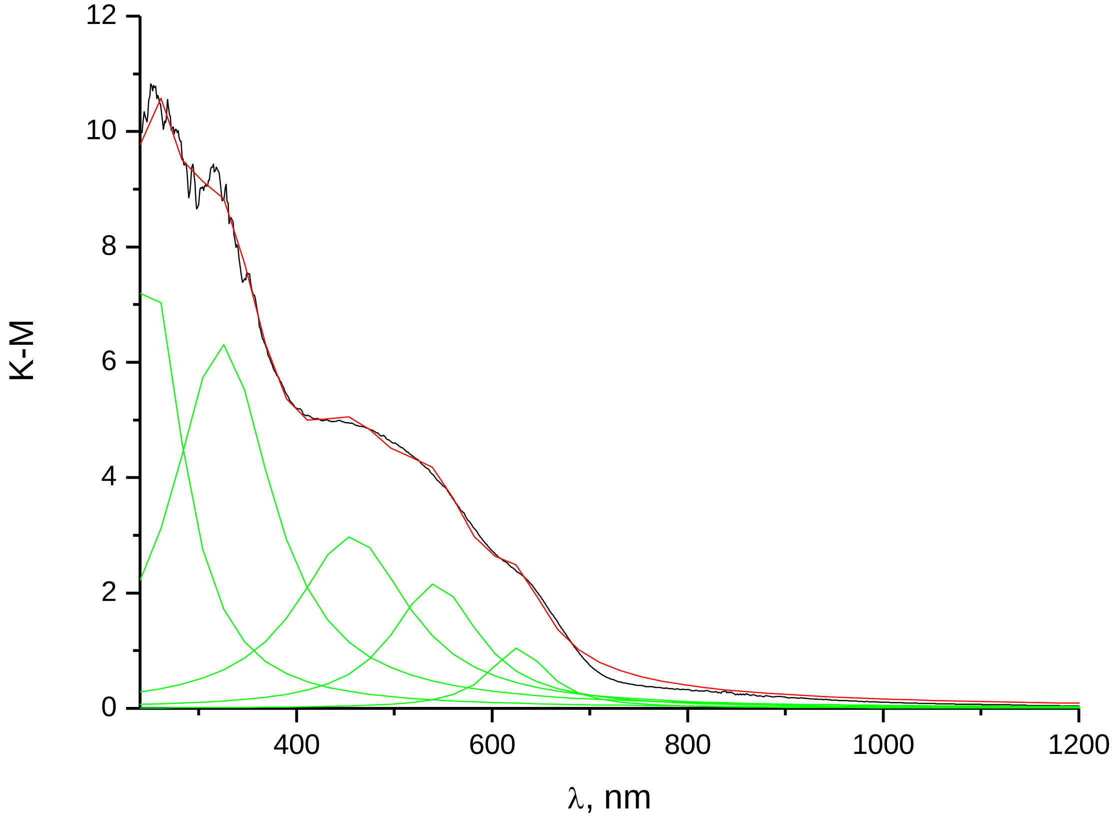

Figure S9. Comparison of the experimental DRS and model for complex $[FeL_2]Br_2{\cdot}H_2O$.

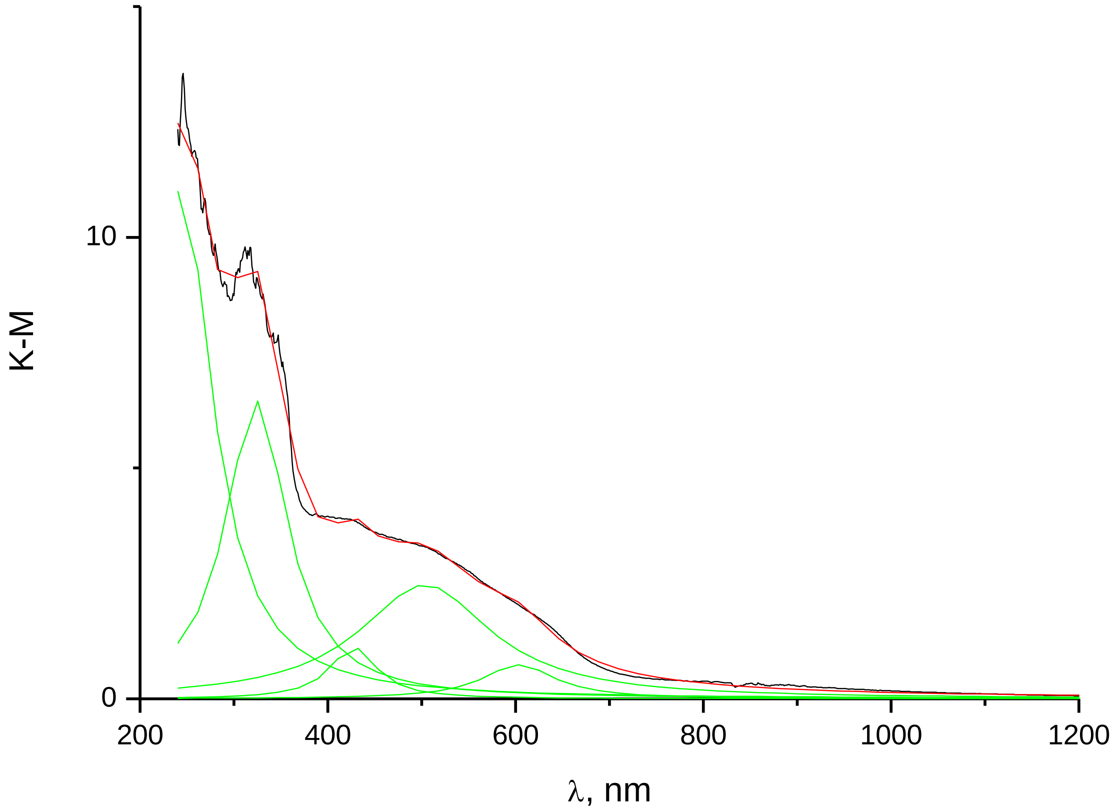

Figure S10. Comparison of the experimental DRS and model for complex $[FeL_2](ReO_4)_2$.

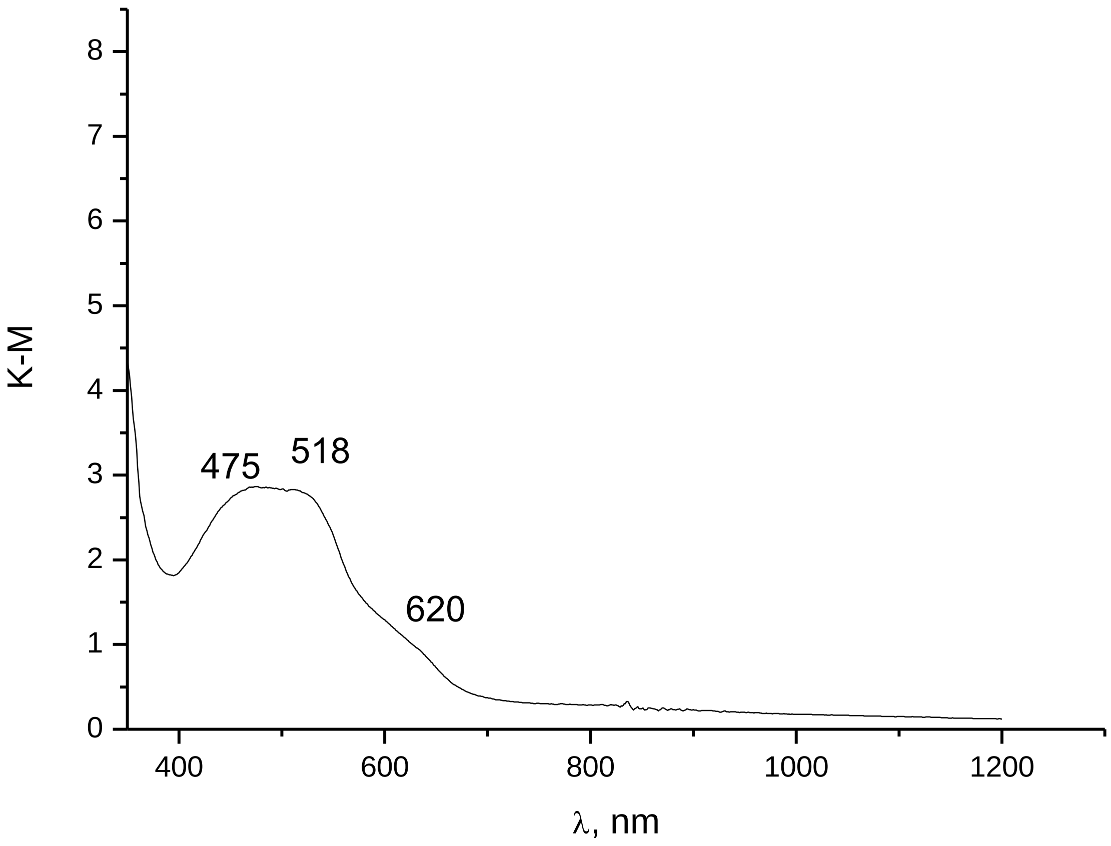

Figure S11. DRS for complex $[FeL_2]B_{10}H_{10}\cdot H_2O$.

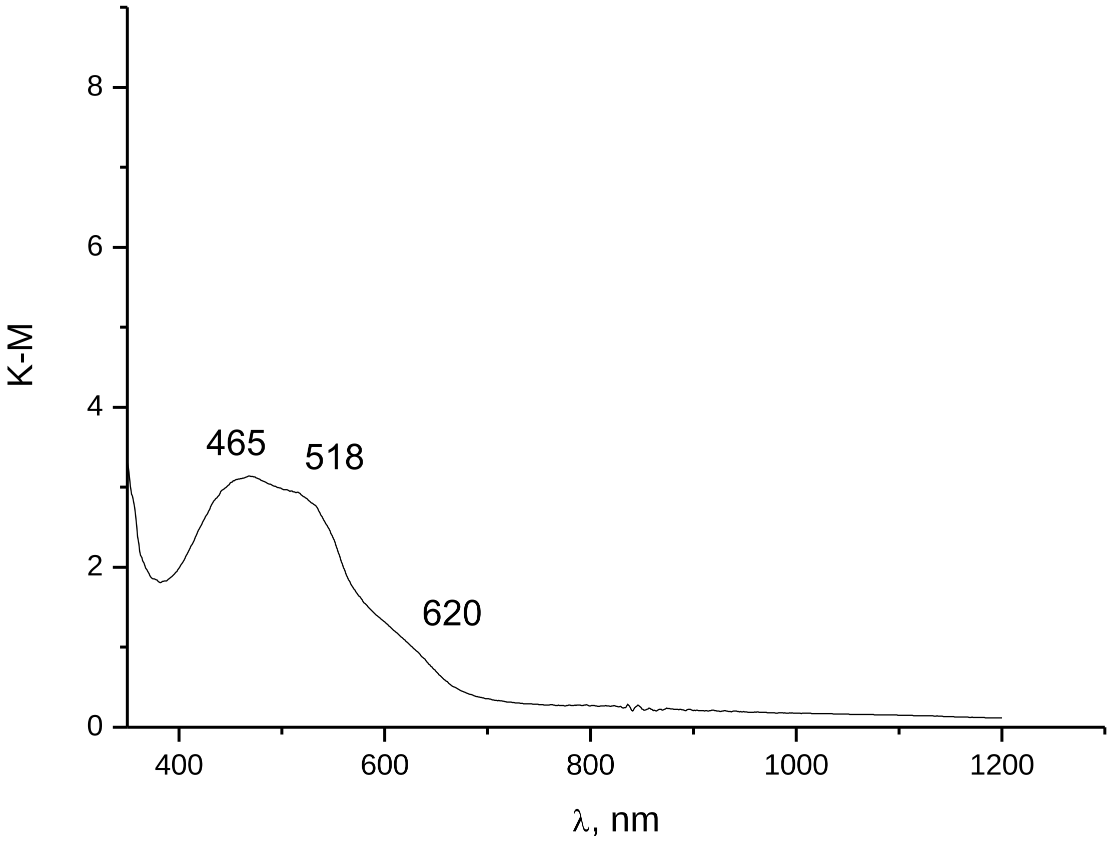

Figure S12. DRS for complex $[FeL_2]B_{12}H_{12}\cdot 1.5H_2O$.
